# A comparison of the dietary patterns derived by principal component analysis and cluster analysis in older Australians

**DOI:** 10.1186/s12966-016-0353-2

**Published:** 2016-02-29

**Authors:** Maree G. Thorpe, Catherine M. Milte, David Crawford, Sarah A. McNaughton

**Affiliations:** Centre for Physical Activity and Nutrition Research, School of Exercise and Nutrition Sciences, Deakin University, 221 Burwood Highway, Burwood, VIC 3125 Australia

**Keywords:** Principal component analysis, Cluster analysis, Dietary patterns, Comparison, Older adults, Retirement

## Abstract

**Background:**

Despite increased use of dietary pattern methods in nutritional epidemiology, there have been few direct comparisons of methods. Older adults are a particularly understudied population in the dietary pattern literature. This study aimed to compare dietary patterns derived by principal component analysis (PCA) and cluster analysis (CA) in older adults and to examine their associations with socio-demographic and health behaviours.

**Methods:**

Men (*n* = 1888) and women (*n* = 2071) aged 55–65 years completed a 111-item food frequency questionnaire in 2010. Food items were collapsed into 52 food groups and dietary patterns were determined by PCA and CA. Associations between dietary patterns and participant characteristics were examined using Chi-square analysis. The standardised PCA-derived dietary patterns were compared across the clusters using one-way ANOVA.

**Results:**

PCA identified four dietary patterns in men and two dietary patterns in women. CA identified three dietary patterns in both men and women. Men in cluster 1 (fruit, vegetables, wholegrains, fish and poultry) scored higher on PCA factor 1 (vegetable dishes, fruit, fish and poultry) and factor 4 (vegetables) compared to factor 2 (spreads, biscuits, cakes and confectionery) and factor 3 (red meat, processed meat, white-bread and hot chips) (mean, 95 % CI; 0.92, 0.82–1.02 vs. 0.74, 0.63–0.84 vs. −0.43, −0.50– −0.35 vs. 0.60 0.46–0.74, respectively). Women in cluster 1 (fruit, vegetables and fish) scored highest on PCA factor 1 (fruit, vegetables and fish) compared to factor 2 (processed meat, hot chips cakes and confectionery) (1.05, 0.97–1.14 vs. −0.14, −0.21– −0.07, respectively). Cluster 3 (small eaters) in both men and women had negative factor scores for all the identified PCA dietary patterns. Those with dietary patterns characterised by higher consumption of red and processed meat and refined grains were more likely to be Australian-born, have a lower level of education, a higher BMI, smoke and did not meet physical activity recommendations (all *P* < 0.05).

**Conclusions:**

PCA and CA identified comparable dietary patterns within older Australians. However, PCA may provide some advantages compared to CA with respect to interpretability of the resulting dietary patterns. Older adults with poor dietary patterns also displayed other negative lifestyle behaviours. Food-based dietary pattern methods may inform dietary advice that is understood by the community.

**Electronic supplementary material:**

The online version of this article (doi:10.1186/s12966-016-0353-2) contains supplementary material, which is available to authorized users.

## Background

Exploring whole dietary patterns, rather than the individual components, has become increasingly important in examining diet and disease relations [1, 2]. With the complex interaction and correlation between nutrients and other food components, dietary pattern analysis has emerged as a more comprehensive assessment of diet [2]. Furthermore, this multi-dimensional and food-based approach can help provide dietary advice that is understood by the community [2]. Three categories of dietary pattern assessment methods exist; theoretical methods, empirical methods and hybrid methods. Theoretical methods, also known as *a priori* methods, assess diet based on prior knowledge and scientific evidence [3] for example, the dietary guideline index [4]. Whereas empirical methods, also known as *a posteriori* methods, use statistical approaches to provide information about existing dietary patterns within the population [5]. Further to these methods are hybrid methods, such as reduced rank regression and partial least squares regression that use a combination of theoretical knowledge and statistical approaches to determine dietary patterns [5].

Principal component analysis (PCA) and cluster analysis (CA) are two commonly applied empirical dietary pattern methods [6]. PCA uses the correlation matrix of food intake variables to identify common patterns of food consumption within the data in order to account for the largest amount of variation in diet [6]. CA groups individuals with similar dietary patterns into mutually exclusive categories according to the mean of the food intake variables [6]. Several CA algorithms exist, with k-means being the most popular in nutrition research because it can handle a large number of input variables efficiently [6].

Both PCA and CA have been extensively used for examining dietary patterns [1, 6, 7], however, they take alternative approaches to addressing the issue. Few studies have directly compared the outcomes of PCA and CA within the same data set [8–16]. Those studies show that both PCA and CA are able to identify comparable key dietary patterns, often identifying a fruit and vegetable dominant pattern vs. a red and processed meat pattern. Comparison studies of dietary pattern methodologies can help us to understand the strengths and weaknesses of their application in nutrition research. However, little research has focused on dietary patterns of older adults with only one known comparison study of PCA and CA [10].

Focusing on health behaviours, such as dietary patterns, among older people has become increasingly important particularly with the ageing population [17]. Prevention campaigns that target diet in older adults may help improve quality of life and reduce morbidity and premature mortality rates [18]. To our knowledge, no studies have explored dietary patterns during the transition period nearing retirement. Peri-retirement, defined as the age of 55 to 65 years, is an important time where major life course transitions occur. Transitional events such as those related to employment, family or health-related circumstances have the potential to impact dietary patterns [19, 20]. Therefore an opportunity exists for public health strategies to be implemented within this population [21]. The objective of the current analysis was to compare dietary patterns derived by PCA and CA and to examine their associations with socio-demographic and health behaviours of a sample of 55 to 65 year old adults.

## Methods

### Participants

This study used data collected as part of the Wellbeing Eating and Exercise for a Long Life (WELL) study. The methods of this study has been described in detail elsewhere [22]. In brief, the WELL study is a longitudinal cohort study with data collected via a postal survey. A random sample of 11,256 Australian adults aged 55–65 years at the census date (31 October 2009) in Victoria were selected from the Australian Electoral Commission’s electoral roll, which is compulsory for Australian citizens to be registered on. A stratified random sampling process was used to select the sample according to sex and socio-economic position [22]. A total of 475 could not be delivered or the participant did not meet the studies age criteria, resulting in 10,781 eligible participants. A total of 4082 volunteers returned surveys, (38 % response rate; 48 % male; 53 and 44 % of males and females respectively had obtained an education level of up to year 12, 20 and 28 % had obtained a trade or certificate qualification and 27 and 28 % had obtained a university degree). Those with complete surveys and sufficient dietary data (having completed at least 90 % of the food frequency questionnaire) were included in this study. Ethical approval to conduct the WELL study was approved by Deakin University Human Research Ethics Committee (2009–105).

### Dietary intake

Dietary intake was assessed using a 111-item Food Frequency Questionnaire (FFQ) adapted from the 1995 Australian National Nutrition Survey [23, 24], based on an existing validated FFQ and has been used in other cohorts in Australia [25–28]. The FFQ assessed participant’s dietary intake over the previous six months, with nine response categories for each item, ranging from ‘never or less than once a month’ to ‘6+ times per day’. No information was gathered on portion sizes. Participants with > 10 % of the FFQ data missing were considered invalid [29] and not included in this study while all other missing FFQ responses were considered not consumed. FFQ responses were converted to daily equivalents and the 111 items were categorised into 52 food groups according to their nutritional content, culinary usage and the 2013 Australian Dietary Guidelines food groups [30], in line with previous dietary pattern studies [2] (Additional file 1: Table S1). FFQ items consumed once per week or more by less than 10 % of the population were combined with other food items where possible or omitted. Only soy beverages were omitted from analyses since a large proportion of the sample (91 %) indicated they never consumed this item. The daily intake frequencies were used to determine dietary patterns as the FFQ did not include portion sizes so grams per day were not available. However servings per day (frequency), is routinely used to determine empirical dietary patterns [6].

### Participant characteristics and health behaviours

Self-reported socio-demographic and health behaviours, including height and weight, were collected in the postal survey. Body mass index (BMI) was calculated and categorized according to the World Health Organization criteria (Underweight: BMI <18.5; Healthy: BMI ≥ 18.5 to < 25 kg/m^2^; Overweight: BMI ≥ 25 to < 30 kg/m^2^; Obese: BMI ≥ 30 kg/m^2^) [31]. Several studies have examined the validity of self-reported height and weight among adults, finding high correlations between self-reported and objectively-assessed weight, including among older adults [32–35].

Self-reported physical activity in the seven days prior to the survey was assessed using the long version of the International Physical Activity Questionnaire (IPAQ) [36]. IPAQ records the frequency, intensity, and duration of leisure time physical activity during the previous week. Minutes of activity per week were calculated by summing the number minutes of moderate intensity physical activity per week and twice the number of minutes per week spent participating in vigorous intensity physical activity per week [36]. Participants were classified as to whether they met the physical activity recommendations of at least 150 min of activity per week [18].

### Statistical analysis

#### Principal component analysis

The food groups were entered into the PCA procedure using the software Stata (StataCorp, Version 12.0). Since the PCA output results in a large number of factor solutions (as many as there are food groups), it is important to identify the key dietary patterns. Firstly, factors with eigenvalues >1.0 were considered, then the break in the scree plot was examined to determine the number of key identified dietary patterns and then the interpretability of the identified patterns was assessed [37]. The identified factors were orthogonally rotated to simplify the factor structure and to enhance their interpretability [38]. For each factor, foods with factor loadings of | ≥ 0.2| were considered to contribute significantly to the pattern and used to calculate factors scores [39]. Factor scores were calculated for each of the derived patterns by summing the products of the observed consumption frequency and the factor loading for each of the significant food groups [40]. Factors were numbered and given provisional labels according to the food groups that loaded highly on the pattern.

PCA was initially conducted separately for men and women and Tucker’s coefficient of congruence was used to assess agreement between sexes [41] to determine if analyses should be stratified by sex. The coefficient of congruence indicated that the dietary factors of men and woman were not similar (data not shown). Therefore all dietary pattern analyses and subsequent tests were stratified by sex.

#### Cluster analysis

K-means CA was employed to determine dietary clusters. Frequency of food intake of the 52 food groups were converted to z-scores (standardised), and entered into the cluster algorithm using the software Stata (StataCorp, Version 12). Standardised food intakes were used to ensure all foods have equal influence on the cluster procedure [7] as cluster analysis is sensitive to outliers [6]. A number of steps were taken to determine the number of identified clusters. Firstly, the Ward’s hierarchical clustering method and the Duda-Hart stopping rule [42] was considered. Secondly, k-means cluster solutions of 2–8 clusters (the range of clusters found previously in the literature [8]) were run using the Calinski-Harabasz stopping rule. These stopping rules examine the between- and within-cluster variance to ensure the most distinct clustering k-means cluster solution is obtained [42, 43]. If a cluster contained <10 % of the total sample it was considered too small for adequate statistical power [44]. Finally, the interpretability of clusters was examined to confirm the final solution. The resulting clusters were numbered and given provisional labels according to the food groups that had a significantly higher mean frequency. Since CA is sensitive to small changes [44] the stability of the final cluster solution was tested. The sample was randomly split in half and the analysis was re-run. Agreement between the clusters of the total sample against a random half was tested with Kappa statistic using standard cut-offs (<0 poor; 0.00–0.20 slight; 0.21–0.40 fair; 0.41–0.60 moderate; 0.61–0.80 substantial; and 0.81–1.00 almost perfect) [45].

Participant characteristics across tertiles of PCA dietary patterns and dietary clusters were explored using Chi-square analysis. The mean PCA factor scores by clusters were compared using ANOVA and a bonferroni post-hoc test. For ease of interpretation, the factor scores were standardised so that the patterns could be compared on the same scale. Data presented in the text are mean and 95 % confidence intervals (CI) unless otherwise specified.

## Results

A total of 3959 (1888 men and 2071 women) participants with complete data were included in this study (Table 1). Compared to men, women were more likely to have a lower BMI, be separated, divorced, widowed or retired, have a lower level of education, were less likely to be smokers and were more likely to be meeting physical activity recommendations (all *P* < 0.001).

**Table 1:**
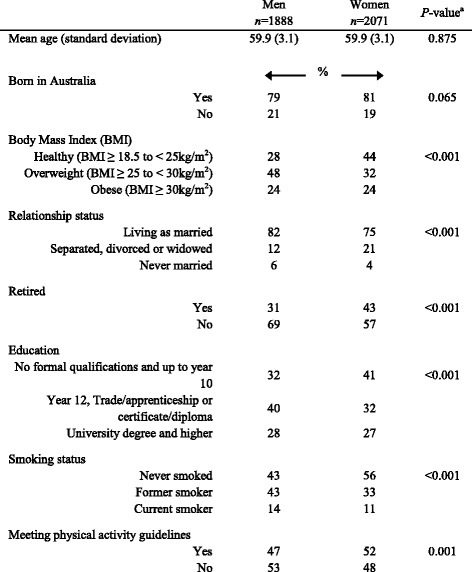
Participant characteristics and health behaviours by sex, Wellbeing Eating and Exercise for a Long Life study 2010

^a^ANOVA and Chi-square analysis

### Principal component analysis

PCA identified four dietary patterns in men and two in women (Table 2). For men, factor 1 was characterised by high factor loadings for vegetable dishes, fruit, fish and poultry with a negative loading for potato. Factor 2 was characterised by high loadings for spreads, biscuits, cakes and confectionery. Factor 3 was characterised by high loadings for red and processed meat, white bread, fried fish and hot chips while having negative loadings for muesli or porridge and reduced fat milk. Factor 4 was characterised by high loadings for a range of vegetables (orange, dark green and cruciferous, potato and other vegetables). These patterns explained 5.8, 5.7, 5.6 and 5.6 % of the variation in food intakes, respectively. In women, factor 1 was characterised by vegetables, fruit and fish and factor 2 was characterised by high loadings for cakes, processed meat, hot chips and confectionery. These patterns explained 7.8 and 6.5 % of the variation in food intakes in women.

**Table 2:** Factor loadings for food groups that loaded highly (| > 0.2|) in varimax rotated principal components for men and women^a^

	Men^b^					Women^c^	
	Factor 1	Factor 2	Factor 3	Factor 4		Factor 1	Factor 2
Eigenvalue	4.4	3.2	2.2	2.0	Eigenvalue	4.2	3.3
% variance explained	5.8 %	5.7 %	5.6 %	5.6 %	% variance explained	7.8 %	6.5 %
Vegetable dishes	0.31	-	-	-	Other vegetables	0.34	-
Fish and other seafood	0.31	-	-	-	Salad vegetables	0.34	-
Oil and vinegar salad dressing	0.31	-	-	-	Vegetable dishes	0.29	-
Salad vegetables	0.28	-	-	-	Dark green and cruciferous vegetables	0.29	-
Rice	0.24	-	-	-	Fruit	0.26	-
Legumes or beans	0.22	-	-	-	Fish and other seafood	0.25	-
Cottage or ricotta cheese	0.22	-	-	-	Orange vegetables	0.25	-
Fruit	0.22	-	-	-	Legumes or beans	0.23	-
Poultry	0.20	-	-	-	Nuts or seeds	0.23	-
Potato	−0.21	-	-	-	Cakes, pastries or other desserts	-	0.27
Spreads and preserves	-	0.34	-	-	Processed or cured meat	-	0.26
Sweet biscuits	-	0.28	-	-	Sweet biscuits	-	0.25
Cakes, pastries or other desserts	-	0.27	-	-	Hot chips, roast potato or wedges	-	0.23
Wholegrain bread	-	0.26	-	-	Chocolate or confectionery	-	0.23
Margarine	-	0.24	-	-	High-energy drinks	-	0.23
Savoury crackers	-	0.23	-	-	Meat pie or sausage rolls	-	0.22
Chocolate or confectionery	-	0.23	-	-	Potato	-	0.21
Cheddar cheese	-	0.22	-	-			
Breakfast cereal	-	0.22	-	-			
Processed or cured meat	-	-	0.29	-			
Pizza and/or Hamburger	-	-	0.28	-			
Red meat	-	-	0.28	-			
White-bread	-	-	0.25	-			
Fried or battered fish	-	-	0.25	-			
High-energy drinks	-	-	0.23	-			
Hot chips, roast potato or wedges	-	-	0.20	-			
Muesli or porridge	-	-	−0.20	-			
Reduced fat milk	-	-	−0.22	-			
Orange vegetables	-	-	-	0.50			
Dark green and cruciferous vegetables	-	-	-	0.44			
Other vegetables	-	-	-	0.44			
Potato	-	-	-	0.36			

^a^Only food groups with factor loadings | ≥ 0.2| are displayed and listed in order for simplicity and easy of interpretation

^b^Male factors; factor 1: vegetable dishes, fruit, fish and poultry; factor 2: spreads, biscuits, cakes and confectionery; factor 3: red meat, processed meat, white-bread and hot chips; factor 4: vegetables

^c^Female factors; factor 1: fruit vegetables and fish; factor 2: processed meat, hot chips cakes and confectionery

### Cluster analysis

The three cluster solution produced the best cluster outcome for both men and women as it formed reasonably sized (>10 % of sample size) and well-separated clusters (determined by a high Calinski-Harabasz pseudo F statistic [43]). The means and standard deviations of the daily food consumption frequency across clusters demonstrated that the identified clustered had varied consumption frequency of key food groups (Table 3). The reliability of the chosen cluster solutions was confirmed by running the analysis on a random 50 % sample, in which the kappa statistic indicated that the random half had good agreement for men (kappa coefficient = 0.72) and very good agreement for women (kappa coefficient = 0.83) in comparison to the total sample (data not shown). Therefore these solutions were considered reliable representations of the dietary clusters in this sample.

**Table 3:** Mean and standard deviation of the daily food consumption frequency of men and women by dietary clusters^a^

	Men^b^			Women^c^		
	Cluster 1	Cluster 2	Cluster 3	Cluster 1	Cluster 2	Cluster 3
Food groups	*n* = 474 (25 %)	*n* = 343 (18 %)	*n* = 1071 (57 %)	*n* = 525 (25 %)	*n* = 409 (20 %)	*n* = 1137 (55 %)
Vegetables and fruit						
Vegetable dishes	**1.85 (1.10)** ^d^	1.25 (0.87)^e^	*1.00 (0.62)* ^f^	**2.42 (1.28)** ^d^	1.46 (0.94)^e^	*1.26 (0.78)* ^f^
Salad vegetables	**2.18 (1.19)** ^d^	1.40 (0.95)^e^	*1.16 (0.72)* ^f^	**2.97 (1.34)** ^d^	1.76 (1.02)^e^	*1.48 (0.80)* ^f^
Dark green and cruciferous vegetables	**1.22 (0.92)** ^d^	0.76 (0.72)^e^	*0.57 (0.47)* ^f^	**1.56 (0.90)** ^d^	0.95 (0.70)^e^	*0.73 (0.55)* ^f^
Orange vegetables	**1.14 (0.70)** ^d^	0.83 (0.59)^e^	*0.61 (0.41)* ^f^	**1.32 (0.73)** ^d^	1.13 (0.66)^e^	*0.76 (0.46)* ^f^
Potato	0.48 (0.39)^d^	**0.62 (0.58)** ^e^	*0.36 (0.28)* ^f^	0.37 (0.34)^d^	**0.64 (0.47)** ^e^	0.34 (0.29)^d^
Other vegetables	**2.05 (1.06)** ^d^	1.50 (0.87)^e^	*1.09 (0.58)* ^f^	**2.50 (1.13)** ^d^	1.73 (0.90)^e^	*1.33 (0.65)* ^f^
Legumes/beans	**0.31 (0.60)** ^d^	0.16 (0.38)^e^	0.12 (0.18)^e^	**0.35 (0.47)** ^d^	0.11 (0.15)^e^	0.13 (0.19)^e^
Fruit	**3.13 (1.86)** ^d^	1.96 (1.73)^e^	*1.58 (1.26)* ^f^	**3.79 (2.05)** ^d^	2.41 (1.51)^e^	*2.11 (1.43)* ^f^
Dried fruit	**0.30 (0.55)** ^d^	0.12 (0.34)^e^	0.09 (0.20)^d^	**0.41 (0.57)** ^d^	0.19 (0.30)^e^	0.15 (0.28)^e^
Nuts and/or seeds	**0.84 (0.89)** ^d^	0.38 (0.43)^e^	0.33 (0.47)^e^	**1.15 (1.06)** ^d^	0.51 (0.64)^e^	*0.37 (0.45)* ^e^
Cereal						
White bread	*0.24 (0.51)* ^d^	**1.22 (1.25)** ^e^	0.40 (0.61)^f^	*0.11 (0.24)* ^d^	**0.52 (0.88)** ^e^	0.25 (0.50)^f^
Wholegrain bread	**1.25 (1.10)** ^d^	0.60 (0.94)^e^	0.56 (0.64)^e^	0.90 (0.88)^d^	0.95 (0.97)^d^	*0.56 (0.61)* ^e^
Savoury crackers	**0.55 (0.73)** ^d^	0.41 (0.56)^e^	*0.24 (0.33)* ^f^	0.43 (0.51)^d^	**0.59 (0.67)** ^e^	*0.30 (0.34)* ^f^
Muesli or porridge	**0.58 (0.84)** ^d^	0.17 (0.34)^e^	0.23 (0.37)^e^	**0.65 (0.72)** ^d^	0.31 (0.40)^e^	0.33 (0.47)^e^
Breakfast cereal	0.53 (0.69)^d^	0.55 (0.77)^d^	*0.40 (0.45)* ^e^	0.37 (0.64)^d^	**0.54 (0.72)** ^e^	0.32 (0.45)^d^
Rice	**0.29 (0.39)** ^d^	0.15 (0.22)^e^	0.19 (0.32)^e^	**0.29 (0.47)** ^d^	0.16 (0.17)^e^	0.17 (0.32)^e^
Pasta	**0.23 (0.20)** ^d^	0.17 (0.35)^e^	0.16 (0.16)^e^	0.19 (0.20)^d^	0.18 (0.36)^d^	*0.14 (0.14)* ^e^
Meat						
Red meat	0.78 (0.52)^d^	**1.13 (0.80)** ^e^	*0.67 (0.44)* ^f^	0.65 (0.56)^d^	**0.92 (0.72)** ^e^	*0.57 (0.39)* ^f^
Processed or cured meat	0.40 (0.36)^d^	**0.75 (0.62)** ^e^	*0.34 (0.31)* ^f^	0.26 (0.37)^d^	**0.47 (0.46)** ^e^	0.23 (0.22)^d^
Poultry	**0.31 (0.28)** ^d^	0.25 (0.25)^e^	*0.19 (0.19)* ^f^	0.34 (0.39)^d^	0.34 (0.32)^d^	*0.23 (0.22)* ^e^
Fish and other seafood	**0.49 (0.47)** ^d^	0.29 (0.27)^e^	0.28 (0.28)^e^	**0.68 (0.62)** ^d^	0.32 (0.37)^e^	0.32 (0.27)^e^
Fried or battered fish	0.07 (0.11)^d^	**0.11 (0.12)** ^e^	*0.05 (0.07)* ^f^	0.06 (0.30)^d^	0.06 (0.08)^d^	0.04 (0.07)^d^
Eggs	0.27 (0.25)^d^	0.30 (0.29)^d^	*0.19 (0.19)* ^e^	0.33 (0.38)^d^	0.29 (0.41)^d^	*0.18 (0.18)* ^e^
Dairy						
Flavoured milk drinks	0.05 (0.14)^d^	**0.26 (0.64)** ^e^	0.06 (0.17)^d^	0.06 (0.23)^d^	**0.10 (0.26)** ^e^	0.06 (0.23)^d^
Whole milk	0.16 (0.42)^d^	**0.69 (1.07)** ^e^	0.18 (0.44)^d^	0.05 (0.23)^d^	**0.16 (0.60)** ^e^	0.09 (0.34)^d^
Reduced fat milk	**0.69 (0.98)** ^d^	*0.26 (0.59)* ^e^	0.39 (0.57)^f^	**0.61 (0.83)** ^d^	0.57 (0.76)^e^	0.45 (0.63)^e^
Cream	0.07 (0.13)^d^	**0.14 (0.38)** ^e^	0.05 (0.10)^d^	0.07 (0.16)^d^	**0.14 (0.41)** ^e^	0.05 (0.12)^d^
Ice-cream	0.20 (0.37)^d^	**0.31 (0.33)** ^e^	*0.15 (0.23)* ^f^	0.12 (0.19)^d^	**0.32 (0.70)** ^e^	0.11 (0.19)^d^
Yoghurt	**0.50 (0.61)** ^d^	0.21 (0.42)^e^	0.19 (0.31)^e^	**0.76 (0.84)** ^d^	0.33 (0.36)^e^	0.38 (0.48)^e^
Cottage or ricotta cheese	*0.08 (0.22)* ^d^	0.03 (0.11)^e^	0.03 (0.09)^e^	**0.17 (0.31)** ^d^	0.03 (0.09)^e^	0.04 (0.11)^e^
Cheddar cheese	0.53 (0.59)^d^	0.59 (0.69)^d^	*0.32 (0.31)* ^e^	0.42 (0.41)^d^	**0.55 (0.55)** ^e^	*0.30 (0.29)* ^f^
Other						
Water	**3.05 (1.98)** ^d^	2.42 (2.01)^e^	*2.06 (1.84)* ^f^	**4.07 (2.00)** ^d^	3.25 (2.02)^e^	3.08 (2.07)^e^
Coffee	1.56 (1.43)^d^	**2.05 (1.78)** ^e^	1.67 (1.50)^d^	1.47 (1.35)^d^	1.43 (1.54)^d^	1.51 (1.43)^d^
Tea	**2.03 (1.59)** ^d^	1.69 (1.73)^e^	*1.42 (1.53)* ^f^	2.08 (1.64)^d^	**2.58 (1.79)** ^e^	*1.77 (1.60)* ^f^
Fruit or vegetable juice	0.48 (0.67)^d^	0.47 (0.64)^d^	*0.29 (0.44)* ^e^	0.34 (0.62)^d^	0.30 (0.47)^de^	0.25 (0.40)^e^
High-joule drinks	0.35 (0.63)^d^	**1.16 (1.40)** ^e^	0.32 (0.50)^d^	0.17 (0.45)^d^	**0.46 (0.90)** ^e^	0.18 (0.42)^d^
Low-joule drink	0.17 (0.46)^d^	0.29 (0.84)^e^	0.22 (0.58)^de^	0.16 (0.45)^d^	**0.36 (0.91)** ^e^	0.21 (0.61)^d^
Beer	*0.35 (0.57)* ^d^	**1.05 (1.71)** ^e^	0.56 (1.06)^f^	0.05 (0.20)^d^	0.04 (0.18)^d^	0.06 (0.38)^d^
Wine	**0.59 (0.83)** ^d^	0.36 (0.61)^e^	0.45 (0.77)^e^	0.52 (0.75)^d^	0.46 (0.82)^d^	0.41 (0.71)^d^
Spirits and liqueurs	0.08 (0.18)^d^	**0.20 (0.58)** ^e^	0.10 (0.26)^d^	0.07 (0.22)^d^	**0.12 (0.45)** ^e^	0.06 (0.21)^d^
Cakes, pastries or desserts	0.39 (0.43)^d^	**0.54 (0.56)** ^e^	*0.21 (0.28)* ^f^	0.22 (0.26)^d^	**0.56 (0.68)** ^e^	0.18 (0.21)^d^
Sweet biscuits	0.37 (0.62)^d^	**0.69 (0.91)** ^e^	*0.22 (0.34)* ^f^	0.19 (0.37)^d^	**0.66 (0.77)** ^e^	0.15 (0.23)^d^
Chocolate or confectionery	0.29 (0.42)^d^	**0.65 (0.81)** ^e^	*0.19 (0.26)* ^f^	0.28 (0.44)^d^	**0.60 (0.81)** ^e^	*0.19 (0.28)* ^f^
Meat pie or sausage rolls	0.05 (0.06)^d^	**0.17 (0.37)** ^e^	0.06 (0.08)^d^	0.02 (0.06)^d^	**0.06 (0.07)** ^e^	0.03 (0.05)^d^
Pizza or Hamburger	0.08 (0.08)^d^	**0.16 (0.17)** ^e^	0.07 (0.07)^d^	0.05 (0.07)^d^	**0.07 (0.07)** ^e^	0.05 (0.06)^d^
Spreads and preserves	0.81 (0.77)^d^	0.79 (0.97)^d^	*0.41 (0.43)* ^e^	0.52 (0.55)^d^	**0.83 (0.72)** ^e^	0.38 (0.43)^d^
Potato chips etc.	0.05 (0.11)^d^	**0.16 (0.26)** ^e^	0.05 (0.11)^d^	0.03 (0.08)^d^	**0.12 (0.35)** ^e^	0.04 (0.08)^d^
Oil and vinegar salad dressing	**0.30 (0.34)** ^d^	0.21 (0.29)^e^	*0.14 (0.21)* ^f^	**0.42 (0.45)** ^d^	0.22 (0.24)^e^	0.20 (0.24)^e^
Creamy salad dressing	0.13 (0.19)^d^	**0.16 (0.25)** ^e^	*0.07 (0.13)* ^f^	0.16 (0.25)^d^	**0.20 (0.29)** ^e^	*0.08 (0.13)* ^f^
Margarine	0.69 (0.89)^d^	**0.98 (1.24)** ^e^	*0.47 (0.59)* ^f^	0.34 (0.53)^d^	**0.98 (1.06)** ^e^	0.36 (0.52)^d^
Butter	0.30 (0.61)^d^	**0.63 (1.02)** ^e^	*0.20 (0.42)* ^f^	0.25 (0.51)^d^	**0.47 (0.74)** ^e^	0.20 (0.35)^d^
Hot chips, roast potato or wedges	0.11 (0.18)^d^	**0.26 (0.39)** ^e^	0.11 (0.13)^d^	0.09 (0.17)^d^	**0.17 (0.21)** ^e^	0.08 (0.10)^f^

^a^All values are mean (SD) unless specified

^b^Male clusters; cluster 1: fruit, vegetables, wholegrain bread, fish and poultry; cluster 2: red meat, processed meat, refined grains and high-energy drinks; cluster 3: small eaters

^c^Female clusters; cluster 1: fruit, vegetables and fish. Cluster 2: red meat, processed meat, cereals and confectionery. Cluster 3: small eaters

^d,e,f^Mean values between cluster without common letter differ, *P*<0.05 tested with ANOVA and bonferroni post hoc. The highest and lowest frequencies for each food group are marked bold and italic, respectively

In men, cluster 1 (*n* = 474) was characterised by higher intake of fruit, vegetables, wholegrain bread, fish and poultry. The men within cluster 2 (*n* = 343) had higher intakes of red and processed meat, white bread, flavoured drinks, cakes, pastries and confectionery. Cluster 3 (*n* = 1071) was characterised by a lower mean frequency for most food items compared to the other clusters and was called ‘small eaters’. In women, cluster 1 (*n* = 525) was characterised by higher mean frequency of fruit, vegetables, nuts, legumes and fish. Cluster 2 (*n* = 409) was characterised by a higher frequency of red and processed meat, white bread, flavoured drinks, cakes, pastries and confectionery. Similar to men, cluster 3 (*n* = 1137) was labelled ‘small eaters’ and was characterised by a consistently lower mean daily intake frequency for the majority of the food items.

### Principal component analysis and p*articipant characteristics*

Men in the highest tertile of factor 1 (vegetable dishes, fruit, fish and poultry) were more likely to have been born outside of Australia, obtained a higher level of education, be non-smokers and meet the physical activity recommendations (Table 4). Men in the highest tertile of factor 2 (spreads, biscuits, cakes and confectionery) were more likely to have been born within Australia. Factor 2 also had a weak u-shaped association with meeting physical activity recommendations (*P* = 0.03). A higher score on factor 3 (red meat, processed meat, white-bread and hot chips) was associated with men who were younger, had a high BMI, a lower level of education and were more likely to be smokers and not meeting physical activity recommendations. Factor 4 (vegetables) was the only PCA pattern associated with relationship status in men, with men living as married more likely to score high on this pattern compared to those separated or never married. Men who also scored high on factor 4 (vegetables) were more likely to have been born in Australia, have a lower level of education and had a weak U-shaped association with meeting physical activity recommendations (*P* = 0.03). None of the male PCA dietary patterns were associated with retirement status.

**Table 4:**
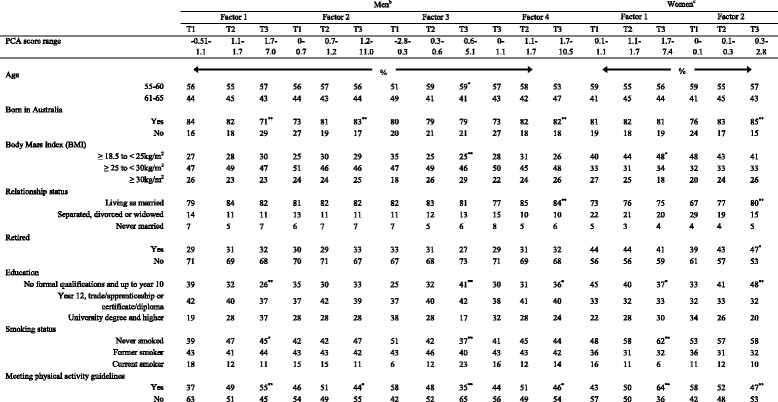
Participant characteristics and health behaviours of men and women according to tertiles of principal component analysis^a^

^a^All tests are Chi square analysis *P < 0.05 **P < 0.001

^b^Male factors; factor 1: vegetable dishes, fruit, fish and poultry; factor 2: spreads, biscuits, cakes and confectionery; factor 3: red meat, processed meat, white-bread and hot chips; factor 4: vegetables

^c^Female factors; factor 1: fruit vegetables and fish. factor 2: processed meat, hot chips cakes and confectionery

For women, a high score on factor 1 (fruit, vegetables and fish) was associated with a lower BMI, a higher level of education, being a non-smoker and meeting the physical activity recommendations (Table 4). Women within the lowest third of factor 2 (processed meat, hot chips cakes and confectionery) tended to be more likely to be born outside of Australia, separated, divorced or widowed, not retired, have a higher education and meeting PA recommendations compared to the middle and highest thirds. No significant associations were shown between PCA dietary patterns and age in women.

### Cluster analysis and p*articipant characteristics*

Men in cluster 2 (red meat, processed meat, refined grains and high-energy drinks) were more likely to be younger and born in Australia compared to the other clusters (Table 5). A higher proportion of men in cluster 2 were classified as obese (BMI ≥ 30 kg/m^2^) and cluster 1 (fruit, vegetables, wholegrains, fish and poultry) had a high proportion of men within the healthy range (BMI ≥18.5 < 25 kg/m^2^). Cluster 1 (fruit, vegetables, wholegrains, fish and poultry) contained men with a higher level of education. Cluster 1 and 3 (small eaters) were more likely to display positive health behaviours (non-smokers and meeting physical activity recommendations) compared to those in cluster 2 (red meat, processed meat, refined grains and high-energy drinks). Relationship status or retirement status of men did not differ between clusters.

**Table 5:**
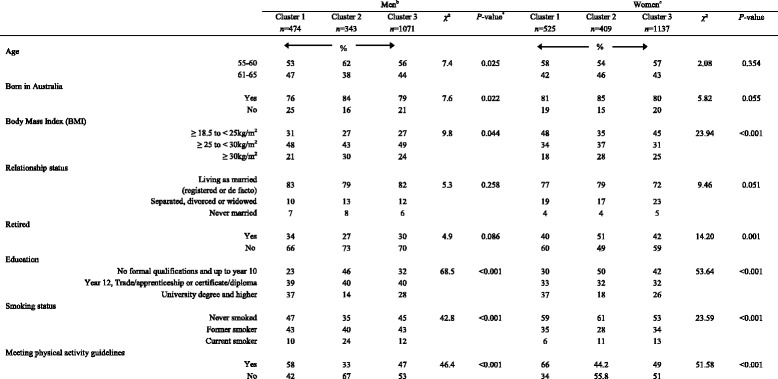
Participant characteristics and health behaviours of men and women according to their dietary cluster^a^

^a^Values are percentages unless otherwise specified

^b^Male clusters; cluster 1: fruit, vegetables, wholegrain bread, fish and poultry; cluster 2: Red meat, processed meat, refined grains and high-energy drinks; cluster 3: Small eaters

^c^Female clusters; cluster 1: fruit, vegetables and fish; cluster 2; red meat, processed meat, cereals and confectionery; cluster 3; small eaters (low mean intake frequency on most items)

The women within cluster 2 (red and processed meat, white bread, flavoured drinks, cakes, pastries and confectionery) were more likely to be overweight (BMI ≥25 < 30 kg/m^2^) or obese (BMI ≥ 30 kg/m^2^) compared to the other clusters. Cluster 1 (fruit, vegetables and fish) contained a high proportion of women within healthy weight range (BMI ≥18.5 < 25 kg/m^2^) (Table 5). Women classified into cluster 2 were more likely to be retired and had achieved a lower level of education compared to the other clusters. Women within cluster 1 (fruit, vegetables and fish) were more likely to be non-smokers and meet physical activity recommendations. No significant differences were found between age, country of birth, and relationship status and clusters in women.

### Comparison of principal component analysis and cluster analysis

**Fig. 1:**
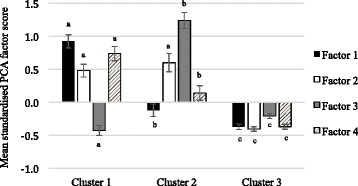
Mean (95 % CI) standarised principal component analysis (PCA) factor scores for each cluster in men. Both factor scores and clusters were derived from the same data set of adults aged 55–65 years participating in the Wellbeing Eating and Exercise for a Long Life study, 2010. Mean values between cluster without common letters differ, *P* < 0.05. Cluster 1: Fruit, vegetables, wholegrain bread, fish and poultry; cluster 2: red meat, processed meat, refined grains and high-joule drinks; cluster 3: small eaters; factor 1: vegetable dishes, fruit, fish and poultry; factor 2: spreads, biscuits, cakes and confectionery; factor 3: red meat, processed meat, white-bread and hot chips; factor 4: vegetables

Men who were grouped into cluster 1 (fruit, vegetables, wholegrain bread, fish and poultry) scored higher on PCA factor 1 (vegetable dishes, fruit, fish and poultry) (mean: 0.92, 95 % CI: 0.82–1.02) and factor 4 (vegetables) (0.74, 0.63–0.84) compared to factor 3 (red meat, processed meat, white-bread and hot chips) (−0.43, −0.50– −0.35) (Fig. 1). Correspondingly, men within cluster 2 (red meat, processed meat, refined grains and high-energy drinks) scored high on factor 3 (red meat, processed meat, white-bread and hot chips) (1.24, 1.12–1.36), followed by factor 2 (spreads, biscuits, cakes and confectionery) (0.60, 0.46–0.74) and scored low on factor 1 (vegetable dishes, fruit, fish and poultry) (−0.12, −0.21– −0.03) and factor 4 (vegetable) (0.14, 0.03–0.25). Men within cluster 3 (small eaters) had negative scores for all four of the PCA patterns. All PCA mean standardized factor scores were significantly different across clusters, except for factor 2 (spreads, biscuits, cakes and confectionery), which did not differ between cluster 1 and cluster 2 (*P* = 0.16).

**Fig. 2:**
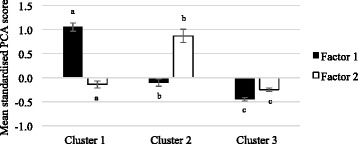
Mean (95 % CI) standarised principal component analysis (PCA)-derived factor scores for each dietary cluster in women. Both factor scores and clusters were derived from the same data set of adults aged 55–65 years participating in the Wellbeing Eating and Exercise for a Long Life study, 2010. Mean values between cluster without common letters differ, *P* < 0.05. Cluster 1: fruit, vegetables and fish; cluster 2: red meat, processed meat, cereals and confectionery; cluster 3: small eaters; factor 1: fruit vegetables and fish; factor 2: processed meat, hot chips cakes and confectionery

Women that were classified into cluster 1 (fruit, vegetables and fish) scored the highest on factor 1 (fruit, vegetables and fish) (1.05, 0.97–1.14) and scored lowest on factor 2 (processed meat, hot chips cakes and confectionery) (−0.14, −0.21– −0.07) (Fig. 2). Women classified into cluster 2 (red meat, processed meat, cereals and confectionery) scored highly on factor 2 (processed meat, hot chips cakes and confectionery) (0.87, 0.73–1.01) compared to factor 1 (fruit, vegetables and fish) (−0.10, −0.18– −0.03). The women in cluster 3 (small eaters) scored low on both the PCA factor 1 and factor 2.

## Discussion

This study demonstrates the comparability between PCA and CA dietary pattern methods with two key dietary patterns (characterised by fruit, vegetables and fish vs. red meat, processed meat and refined grains) identified in both men and women. These dietary patterns are consistent with those previously described in the literature [6], and showed associations with key socio-demographic variables and health behaviours. These results are consistent with previous comparison studies among adults [8–16] and older adults [10], however this study is the first to explore dietary patterns in this specific transitional life stage among Australian adults.

Although this study demonstrated consistencies in the identified dietary patterns, some differences were also acknowledged in the outcome from each method. Using PCA, more dietary patterns were identified in men (4) than women (2), perhaps indicating greater variation in dietary intake in men than women of this age group. The PCA-derived factor 2, characterised by spreads, biscuits, cakes and confectionery identified in men, however a corresponding pattern was not evident in CA. There was no difference in the mean scores for this factor across clusters in men, suggesting that all men shared these snacking-type dietary characteristics of factor 2.

In both men and women, the largest cluster identified (small eaters) was characterised by low consumption frequencies for most food groups relative to the other clusters and it contained no dominating food groups. No equivalent pattern was identified in PCA analysis, perhaps as PCA is driven by correlations between input variables (food frequency) rather than the absolute input values. A similar dominating smaller eaters pattern has been described in other studies among older adults aged 65 years and over [46–48] and in adults aged 18 to 64 years [13]. While it has been suggested in other studies that those in the small eaters cluster might be at risk of malnutrition [47], there is no evidence that the small eaters in the current study are at risk of malnutrition. The small eaters cluster’s mean BMI was 27.7 kg/m^2^ in men and 26.9 kg/m^2^ in women and only 0.3 % were considered underweight (BMI < 18 kg/m^2^). It is possible that this cluster reflects under-reporting, although poorly completed questionnaires were excluded prior to analysis. Furthermore, with respect to under-reporting we would have expect to observe low consumption frequencies for ‘unhealthy’ food groups (e.g., red meat, processed red meat, refined grain) and relatively high consumption frequencies for ‘healthy’ food groups (e.g., fruits, vegetables, fish), which may result particularly from social desirability bias. However, this was not the case in our study. It is also possible that those within the small eater cluster were consuming larger portions but still less frequently than the other clusters. Unfortunately, portion size was not measured in this study, so we cannot investigate the plausibility of this hypothesis. Another possibility is that those in the small eaters cluster have an increased diet variety, consuming low frequencies of many food groups. The authors compared these clusters with adherence to the Australian Dietary Guidelines [30] in further analyses and found that the small eaters cluster did not demonstrated a higher diet variety, however they did demonstrate higher compliance with the guidelines overall compared to those in cluster 2 (red meat, processed meat, refined grains and high-joule drinks), indicating better diet quality (unpublished results).

Each dietary pattern method has individual strengths and weaknesses. Although CA is good at identifying sub-populations with similar characteristics, it may not always be optimal for looking at the relationship between dietary patterns and health outcomes. The statistical power is limited by the need to use a reference category [49] and the uneven cluster sizes of the clusters identified in this study limit the power for future analyses. Furthermore, the limited interpretability of these clusters makes it difficult to translate results into practice. In addition, the continuous nature of the PCA factors is advantageous, since they can be assessed as a continuous variable within a regression model and appear more useful in future analyses in the sample.

Our results demonstrate associations with participant characteristics consistent with the current literature. Previous studies in older adult populations (55y+) have found that vegetable-based diets and those consistent with dietary guidelines are associated with being female, a younger age, a higher level of education, physical activity, a higher BMI and not smoking compared to a meat and processed food-type diet less consistent with dietary recommendations [10, 50]. Dietary patterns of this nature have also been associated with increased nutritional status, quality of life and decreased mortality in older adults [51–55].

Due to the small age range (55–65y) we did not expect to find significant relationships between age and dietary patterns. However, we did show that the younger men were more likely to have dietary patterns characterised by red meat, processed meat, white-bread and refined grains. There are mixed results regarding age and dietary patterns [9, 10], and confounding factors such as cultural and social factors may influence the differences in dietary patterns across age between studies.

We showed that men and women with dietary patterns characterised by red meat, processed meat, white-bread and refined grains were more likely to be overweight and obese compared to those whose dietary patterns consisted of high fruit and vegetables consistent with previous results [56]. However, associations between BMI and dietary pattern have been inconsistent across studies [57, 58]. The disparities in results may be a result of the heterogeneous samples characteristics, limitations in dietary pattern measures and the limited ability to determine causality in observational studies.

Our results show a significant association between PCA dietary patterns and relationship status. Women who were married were more likely to have a dietary pattern characterised by processed meat, hot chips cakes and confectionery compared to those who were separated, while married men were more likely to score high on the vegetable pattern compared to those separated. However, relationship status was not associated with clusters. There is limited and inconclusive research available around marital status and dietary patterns [59]. Previous evidence suggests that those living solitary are more likely to have poorer dietary patterns [60–62]. In a longitudinal study improvements in dietary behaviours over 21 years in women were demonstrated whether they remained married or became single [63]. Another longitudinal study demonstrated remarriage or cohabitation had a positive effect on diet while marital break-up had adverse effects on diet and other health behaviours [19]. The barriers to healthy eating may differ by sex, which is important to acknowledge in public health initiatives. Further research in this area is required as living alone may negatively effect diet contributing to poor health outcomes in these individuals [62].

Retirement status was a significant covariate of dietary patterns for women, but was not important in men. Women who were retired were more likely to have dietary patterns characterised by red and processed meat and refined grains, compared to their non-retired counterparts whose dietary patterns were likely to be characterised by fruit, vegetables, fish and poultry. However, our results are at odds with a previous longitudinal study that found retired women tended to improve dietary patterns post retirement [64]. A review of the evidence on changes in lifestyle behaviours during the transition to retirement concluded that both positive and negative changes occur dependent on the personal circumstances of the retiree [21], but there is not enough evidence to draw any conclusions on changes in dietary habits [21]. A prospective study compared nutritional patterns 6 months before retirement and 18 months after retirement and found that nutrient consumption did not change after retirement however, there were changes in food-related behaviours such a taking more time for breakfast and lunch, eating out more and having guests for meals more frequently [65]. These social changes among other factors such as presence or absence of illness may play a role in influencing dietary pattern among retirees.

Lower levels of education, a measure of socio-economic position, were associated with poorer dietary patterns in this study, consistent with previous research in adults [56, 66–68]. Unfortunately, in the current study, substantial missing data on income (16 %) restricted further investigation of socio-economic position. The relationship between socio-economic position and diet is complex and the drivers of this relationship are not fully understood. A review on socio-economic position and diet quality highlights that most studies focus on lack of knowledge, cooking skills and motivation, in those with lower levels of education accounting for poorer dietary intakes [69]. However, there is little evidence to confirm these theories since the relationship between socio-economic position and dietary intake is multifactorial [69]. Missing data is a common occurrence with relation to sensitive information such as income [70].

Poor diet, smoking and low physical activity are key independent risk factor for chronic diseases [1, 18, 71] Consistent with previous studies, those with poorer dietary patterns (charaterised by red and processed meat and refined grains as opposed to fruit and vegetables) were more likely to be smokers and have lower levels of physical activity [4, 16, 68]. This may identify a group of at risk older adults who demonstrate a cluster of poor health behaviours.

Possible limitations of this study should be considered. No causal relationships could be determined due to the cross-sectional design of this study and the study relied on self-reported measures, which may result in measurement error, for example height, weight and BMI. However, self-reported height and weight has previously been shown to be a valid estimate of BMI in large epidemiological studies [32, 33, 72].

Empirically-based dietary pattern techniques have inherent limitations for dietary pattern analysis. Several researcher-determined decisions are required such as the collapsing and format of input variable, the number of derived patterns and assigning labels for example [6, 7]. In the current study, steps were taken to reduce such subjectivity. For example, the FFQ foods were grouped based on approaches used in previous literature and consistent with the Australian Dietary Guidelines [30]. Established criteria and best practice were used to determine the dietary patterns and objective criteria were used to compare the dietary patterns between men and women in PCA. The use of FFQs are known to be susceptible to measurement error of dietary intake, however other methods such as food records or 24-h recalls would have substantially increased subject burden. This FFQ used in this study has previously been used to assess dietary patterns and behaviours, demonstrating that it is a valid predictor of health outcomes and suggesting it has predictive validity [28, 52, 4, 73].

A limitation of the FFQ used is that it did not measure portion sizes and therefore energy intake could not be estimated [74] and input variables for dietary pattern analysis could not be adjusted for energy intake. However, non energy-adjusted frequency is more sensitive to the intake of important low-energy foods such as fruit and vegetables [5, 8, 75] and previous research has questioned the need for energy adjustment [75–77]. There is conflicting evidence regarding best practice and adjusting for energy may have different implications for different dietary pattern assessment techniques [13].

Strengths of this study include the population-based design of the WELL study, the focus on older adults and the comparison of different methods. Although the response rate was modest (38 %), the sampling technique resulted in a large sample with characteristics consistent with both state [78] and national data [79, 80]. For example, at baseline the WELL study participants had similar levels of employment in comparison to national figures (60 vs. 61 % in full time or part time employment) and they were more highly educated (28 vs. 19 % had completed a university degree or higher). Participants were less likely to be overweight or obese in comparison to national data (64 vs. 74 %) and were less likely to be current smokers (12 vs. 15 %) [80–82]. A similar proportions of the WELL sample were meeting fruit (10 vs. 11 %) and vegetable (61 vs. 56 %) recommendations compared to the national population of the same age [82]. Furthermore, the specific age range of 55–65 years captures an understudied population during a transitional life stage and the comparative nature of this study adds to the limited research in this area. Of the studies that have compared PCA and CA, they have concluded that although the dietary assessment methods are different, the dietary patterns identified often have similar qualities including a fruit and vegetable dominant pattern vs. a red and processed meat pattern [49]. In order to enhance the understanding of the dietary patterns identified in this study population, validation against health outcomes or clinical markers of disease would be advantageous [49].

## Conclusion

Both PCA and CA identified two key dietary patterns in peri-retirement aged men and women. These results add to the limited literature on dietary patterns in older adults. Overall, PCA identified dietary patterns that were more interpretable than CA. This study showed that those with poor diets tend to also display negative health behaviours including smoking and not meeting physical activity recommendations, initiatives targeting these collective health behaviours, which are risk factors for chronic disease, may help to improve the health of older adults.

## Additional file

Additional file 1: Table S1. List of the 52 food groups derived from the 111 items in the food frequency questionnaire. (PDF 81 kb)

## Abbreviations

ANOVA: analysis of variance
BMI: body mass index
CA: cluster analysis
CI: confidence interval
FFQ: Food Frequency Questionnaire
IPAQ: International Physical Activity Questionnaire
PCA: principal component analysis
WELL: Wellbeing Eating and Exercise for a Long Life study
