# Supplementary material for: A comparison of the dietary patterns derived by principal component analysis and cluster analysis in older Australians
Source: Int J Behav Nutr Phys Act. 2016 Feb 29;13:30. doi: 10.1186/s12966-016-0353-2 (PMC4772350; doi:10.1186/s12966-016-0353-2)
Supplement: Additional file 1: Table S1. — List of the 52 food groups derived from the 111 items in the food frequency questionnaire. (PDF 81 kb) [file 12966_2016_353_MOESM1_ESM.pdf]

Additional table 1 List of the 52 food groups derived from the 111 items in the food frequency questionnaire

Food groups	Food items
**Vegetables and fruit**	
Vegetable dishes	Green/mixed salad (including lettuce, tomato etc) in a sandwich; as a side salad/with a main meal; stir-fried or mixed vegetables; vegetable casserole
Dark green and cruciferous vegetables	Silverbeet or spinach; broccoli; cauliflower; brussels sprouts, cabbage or coleslaw
Orange vegetables	Pumpkin; sweet potato; carrots
Salad vegetables	Capsicum; celery or cucumber; tomato; lettuce
Potato	Potato, boiled, mashed or baked
Other vegetables	Peas; green beans; zucchini, eggplant or squash; mushrooms; onion or leeks; sweetcorn/corn on the cob
Legumes or beans	Soybeans or tofu; baked beans; other beans (e.g. chichpeas), lentils
Fruit	Apple or pear; orange, mandarin or grapefruit; bananas; peach or nectarine; plum or apricot; mango or paw paw; pineapple; grapes; melons; strawberries or other berries
Dried fruit	Dried fruit
Nuts or seeds	Peanuts, peanut butter, other nut spreads; other nuts (e.g. almonds, walnuts); seeds (e.g. sunflower, tahini)
**Cereal**	
White bread	White breads, toast or rolls; english muffin, bagel or crumpet
Wholegrain bread	Wholemeal, mixed grain bread, toast or roll
Savoury crackers	Dry or savoury biscuits, crisp-bread, crackers
Muesli or porridge	Muesli, cooked porridge
Breakfast cereal	Breakfast cereal
Rice	Rice (white or brown)
Pasta	Pasta (including filed), noodles
**Meat**	
Red meat	Beef, veal (roast, chop or steak); lamb (roast, chop or steak); pork (roast, chop or steak)[2], mince dishes (e.g. rissoles, meatloaf);mixed dishes with beef, veal, lamb, pork (e.g. casserole, stir-fry); mixed dishes with pork (e.g. casserole, stir-fry), liver (including pate); other offal (e.g. kidneys)
Processed or cured meat	Sausage, frankfurter; bacon; ham; luncheon meats, salami
Poultry	Chicken, turkey, duck (roast, steamed, bbq), mixed dishes with chicken, turkey, duck (e.g. casserole, stir-fry)
Fish and other seafood	Canned fish (e.g. tuna, salmon, sardines); fish (steamed, baked, grilled); other seafood (e.g. prawns, squid)
Fried or battered fish	Fried or battered fish
Eggs	Eggs
**Dairy**	
Flavoured milk drinks	Flavoured milk drinks (e.g. milkshakes, iced coffee, hot chocolate)

Supplementary table 1 List of the 52 food groups derived from the 111 items in the food frequency questionnaire *(continued)*

**Food groups**	**Food items**
Whole milk	Milk as drink; milk on breakfast cereals; milk in hot beverage[1]
Reduced fat milk	Milk as drink; milk on breakfast cereals; milk in hot beverage[1]
Cream	Cream or sour cream
Ice-cream	Ice-cream
Yoghurt	Yoghurt (plain or flavoured)
Cottage or ricotta cheese	Cottage or ricotta cheese
Cheddar cheese	Cheddar and other cheeses
**Other**	
Water	Water
Coffee	Coffee
Tea	Tea
Fruit or vegetable juice	Fruit juice (100% juice); vegetable, tomato juice
High-joule drinks	Fruit juice drink or fruit drink; cordial; soft drink (including flavoured mineral water)
Low-joule drink	Low-joule cordial; low-joule soft drink
Beer	Beer (low alcohol); beer (ordinary)
Wine	White wine or champagne, sparkling wine; red wine; wine cooler
Spirits and liqueurs	Sherry, port, fortified wines; spirits, liqueurs
Cakes, pastries or other desserts	Cakes, sweet muffins, scones or pikelets; sweet pies or sweet pastries; other puddings or desserts
Sweet biscuits	Plain sweet biscuits; cream, chocolate biscuits
Chocolate or confectionery	Chocolate (including chocolate bars e.g. Mars bars); other confectionery (e.g. Sweets or lollies)
Meat pie or sausage rolls	Meat pies, sausage roll or other savoury pastries
Pizza and/or hamburger	Pizza; hamburger
Spreads and preserves	Jam, marmalade, honey or syrups; Vegemite, Marmite or Promite
Potato chips etc	Potato chips, corn chips, Twisties etc
Oil and vinegar salad dressing	Oil and vinegar salad dressing
Creamy salad dressing	Mayonnaise or other creamy dressing
Margarine	Margarine on bread or cooked vegetables
Butter	Butter on bread or cooked vegetables
Hot chips or roast potato	Hot chips, roast potato or potato wedges

1. Participants indicated the type of milk that they usually drink in an addition behaviour question
2. Although pork is not classed as red meat for marketing purposes in Australia, the Australian Dietary Guidelines treat pork as red meat since pork is classified as red meat in the international literature, therefore for the purpose of this work pork will be classed as red meat.
